# *Pisum sativum* Response to Nitrate as Affected by *Rhizobium leguminosarum*-Derived Signals

**DOI:** 10.3390/plants11151966

**Published:** 2022-07-28

**Authors:** Laure Boeglin, Marie-Christine Morère Le-Paven, Thibault Clochard, Joëlle Fustec, Anis M. Limami

**Affiliations:** 1 Univ Angers, INRAE, IRHS, SFR 4207 QuaSaV, 49000 Angers, France; l.boeglin@groupe-esa.com (L.B.); lepaven@univ-angers.fr (M.-C.M.L.-P.); thibault.clochard@univ-angers.fr (T.C.); 2 USC LEVA, École Supérieure des Agricultures, INRAE, SFR 4207 QuaSaV, 49007 Angers, France; j.fustec@groupe-esa.com

**Keywords:** rhizobia, nitrate, signaling, root branching, root foraging, nodules

## Abstract

Legumes are suitable for the development of sustainable agroecosystems because of their ability to use atmospheric N_2_ through symbiotic nitrogen fixation (SNF). However, a basic NO_3_^−^ input is necessary before SNF takes place to ensure successful seedling establishment. Since *Rhizobia* not only induce nodulation but also affect root branching by stimulating the development of lateral roots, and NO_3_^−^ as a signal also modulates root system architecture, we investigated whether *Rhizobium*-derived signals interfere in nitrate signaling. Here, we bring evidence that (i) *Rhizobium*-altered NO_3_^−^-mediated processes in pea expressions of major players in NO_3_^−^ transport, sensing, and signaling were affected, and (ii) the characteristic limitation of root foraging and branching in response to NO_3_^−^ supply was abolished. The number of tertiary roots per secondary root was higher in infected compared to uninfected peas, thus indicating that the *Rhizobium* effect allows for favorable management of trade-offs between nodules growth for nitrogen capture and root foraging for water and other nutrient uptake in pea. The outcome of this basic research can be used to produce molecular tools for breeding pea genotypes able to develop deep-foraging and branched root systems, and more competitive architectures and molecular levels for soil NO_3_^−^ absorption during seedling establishment without jeopardizing nodulation.

## 1. Introduction

Development of sustainable agroecosystems is becoming necessary for protecting soils from pollution caused by the massive usage of chemical fertilizers [1]. For this aim, legumes have been used for their ability to withstand low N input systems because of their capacity to use atmospheric N_2_ through symbiotic nitrogen fixation (SNF) with a further benefit that once established, they progressively fertilize the soil [2]. Although annual legumes remain major objectives for breeders, adaptation to sustainable cropping systems, including ecological services, implies the need for definition of new ideotypes for developing innovative genotypes. Nowadays, including legume-based intercrops in crop successions is gaining renewed interest, as it is considered to be a promising way to ensure a double benefit, namely, decreasing the usage of both N fertilizer and herbicides on top of maintaining good levels of production [3]. Intercropping, with legumes used as service plants, can help control weeds by competing for soil water, mineral nutrients, and light [4]. However, legumes are known to be poor competitors for the soil mineral N, and more efficient genotypes would be needed to improve weed control in innovative sustainable cropping systems. Indeed, a basic input of N (nitrate) fertilizer in the early post-germination stage and before SNF starts is unavoidable to ensure successful seedling establishment of the legume. Therefore, phenotypic traits related to root architecture, such as deep-foraging, fast-growing, and highly branched root systems are critical for legumes regarding soil exploration and efficient mineral nutrient use. Paradoxically, development of these traits might be counteracted by exogenous nitrate acting as a signal molecule with effects on developmental plasticity of root system architecture [5,6]. This is because nitrate does not serve only as a nutrient in plants, but it is also sensed as a signal molecule involved in at least two types of actions: (1) a short term signaling role, called the primary nitrate response (PNR), which results in hundreds of genes transcriptionally regulated by nitrate in a few minutes after its application; and (2) a long term signaling role as evidenced by its effect on root architecture via local and systemic signaling pathways [7,8]. For example, it has been shown that under high NO_3_^−^ supply, primary root growth is partially inhibited in the legume *Medicago truncatula* [9], and lateral root (LR) elongation is systemically inhibited right after their emergence from the primary root in *Arabidopsis thaliana* [10]. 

Furthermore, in the particular case of legumes, competition between nodules and lateral root formation has been described, as nodules and lateral roots both derive from the same structure, namely, de-differentiated root pericycle cells [11]. Stimulation of nodule formation by Nod factors occurs via a complex signaling pathway involving several hormones, e.g., ABA, auxin, cytokinine, and ethylene [12]. Interestingly, in *M. truncatula*, Nod factor application on uninfected roots stimulated lateral root development through the same signaling pathway as that involved in nodule formation [13]. Nod factor treatment did not stimulate lateral root formation in mutants affected in at least one of the key nodulation genes: *NFP*, *DMI1*, *DMI2*, *DMI3*, or *NSP*. This result shows that lateral root formation shares, at least partially, the Nod factor-stimulated symbiotic signaling pathway [13].

It appears then that nitrate and Nod factors are two important determinants of root architecture, in particular in root branching. Nitrate acts as a signal molecule that shapes the root architecture via a signaling pathway involving changes in hormone (auxin, ABA, cytokinine) balance and de-differentiation of root pericycle cells. Considering this, our question is how the presence of Rhizobia may modify the response to nitrate signal. We hypothesize that the presence of Rhizobia via secreted molecules, and probably not only Nod factors, would interfere with the nitrate signaling pathway and finally modify the response to the nitrate signal. Rhizobia have been shown to produce metabolites that may interact with pathways of plant hormonal biosynthesis and signaling, since some Rhizobia strains were shown to synthesize hormones such as auxin and cytokinin [14,15]. 

The aim of the present article is to analyze this hypothesis in the *Pisum sativum*–*Rhizobium leguminosarum* symbiotic system under various nitrate concentrations during early post-germination growth and seedling establishment. In order to control accurately nitrate concentrations, we opted to use either solid, inert support or hydroponics, given that discoveries done under these conditions proved to hold true when plants are grown under field conditions (for a review, see [16]). Pea is suitable for this approach, because compared with the model legumes *M. truncatula* and *Lotus japonicus*, it has been domesticated and bred for agricultural cropping [17], and a reference pea genome that provides information necessary to identify gene families is now available [18]. Furthermore, pea has been shown to be one of the most relevant legume species for weed control at crop settlement when intercropped with rapeseed [4]. Among legume crops, pea is one of the most effective species for soil nitrogen uptake, with a good ability to produce lateral roots (LRs), whatever the level of soil nitrogen availability [19]. The evaluation of our hypothesis will include analyses of molecular (expression of genes) and phenotypic traits known to be sensitive to NO_3_^−^ signaling. 

## 2. Results

### 2.1. Effect of Nitrate in Presence or Absence of Rhizobium on Molecular Traits 

To ensure that *Rhizobium* action via secreted signaling molecules, referred to herein as *Rhizobium*-derived signals, was effective in hydroponics systems, i.e., in the absence of exogenous nitrate (0 mM) and under low (1 mM) or high (10 mM) NO_3_^−^ concentrations, we measured the expressions of genes involved in the nodulation process, which are known to be responsive directly or indirectly to Nod factors. As to the main topic of this study, investigation of the effect of *Rhizobium* on the nitrate signaling pathways at the molecular level, we measured the expressions of nitrate-inducible genes under various nitrate concentration, as mentioned above, in the presence or absence of *Rhizobium* by real-time quantitative PCR. For some genes, expression was also followed by the semi quantitative RT-PCR method.

For this aim, sequences of genes determined in our previous studies as being nitrate-inducible in *M. truncatula* roots at early stages of development [9,20,21,22] were used to browse the *P. sativum* genome database (https://www.pulsedb.org/ (accessed on 8 June 2022)) and search for sequences of their orthologs in this species. Chosen genes were: *MtNR1* (nitrate reductase), *MtGS1* (cytosolic glutamine synthetase), *MtGS2* (plastidic glutamine synthetase), *MtASNS* (asparagine synthetase), *MtNPF6*.*7* (NRT1/PTR Family 6.7), and *MtNAR2*/*MtNRT2* (two component of high affinity nitrate transport system, NAR2/NRT2). Sequences of *M. truncatula* genes involved in nodulation were, similarly as described above, used for the determination of sequences of their orthologs in *P. sativum*. Chosen genes were: *MtNSP1* (nodulation signaling pathway), *MtHAP2.1* (transcription factor of the CCAAT-binding family), and *MtNPF7.6* (NRT1/PTR Family 7.6). 

#### 2.1.1. Markers of the Effect of Rhizobium-Derived Signal

**Figure 1:**
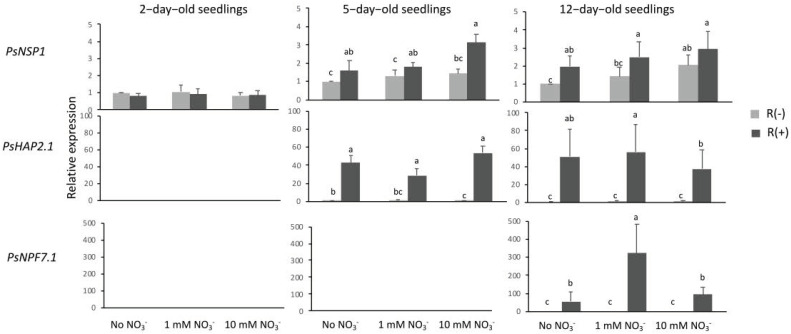
Relative expression of genes involved in the nodulation process. RT-qPCR experiments were performed with root RNAs from 2-, 5-, or 12-day-old seedlings grown in MS without NO_3_^−^ or with 1 mM or 10 mM NO_3_^−^, with [R(+)] or without *Rhizobium* [R(–)] using actin and *β*-tubulin as reference genes. *PsNSP1*, nodulation signaling pathway; *PsHAP2.1*, transcription factor of the CCAAT-binding family; *PsNPF7.1*, (NRT1/PTR Family 7.6). Data presented are means of three independent experiments performed in triplicate. Error bars indicate SE. Letters denote significant differences at the 0.05 level (multiple comparisons).

**Figure 2:**
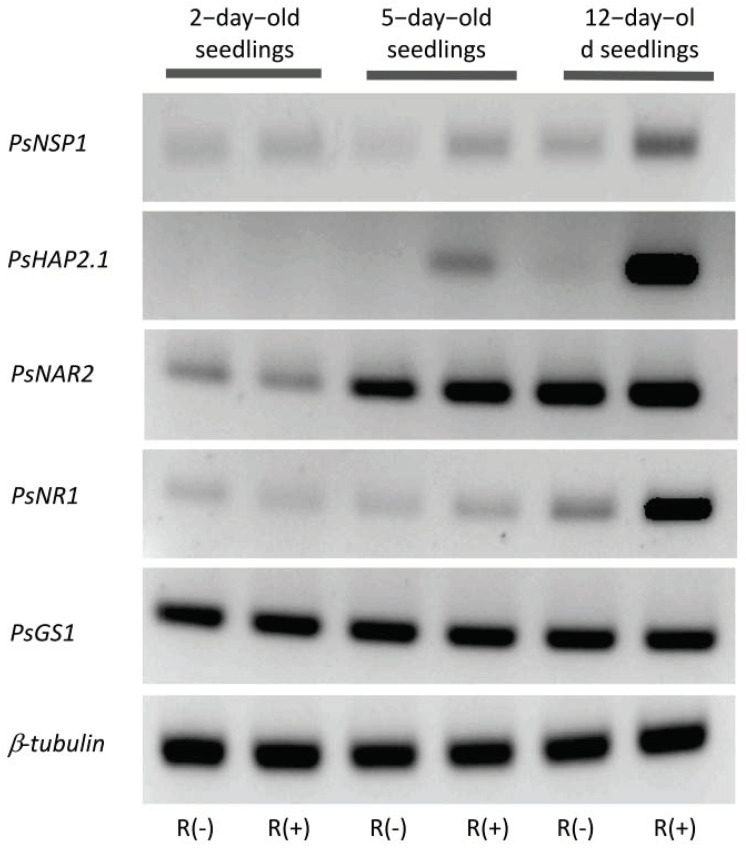
Expression of genes using the semi-quantitative RT-PCR method. Semi-quantitative RT-PCR were performed with root RNAs from 2-, 5-, or 12-day-old seedlings grown in MS without NO_3_^−^ without [R(−)] or with [R(+)] *Rhizobium*. *PsNSP1*, nodulation signaling pathway; *PsHAP2.1*, transcription factor of the CCAAT-binding family; *PsNAR2*, one component of the high-affinity nitrate transport system; *PsNR1*, nitrate reductase; *PsGS1*, cytosolic glutamine synthetase. *β-tubulin* was used as a control.

*NSP1* encodes in legumes a transcription factor expressed in the epidermis as a member of a signaling pathway induced by Nod factors responsible for the expression of ENOD (early nodulin) genes [12]. Mutants affected in *NSP1* expression failed to achieve the infection process. In our conditions, *PsNSP1* showed a basic and constitutive expression (expression in absence of nitrate and *Rhizobium*-derived signals) at all three stages of development (Figure 1). However, in 5- and 12-day-old seedlings, expressions of this gene were all significantly higher in the presence of *Rhizobium* than that in its absence, indicating a specific effect of *Rhizobium*-derived signals (Figure 1 and Figure 2). 

*MtHAP2.1* encodes a transcription factor of the CCAAT-binding family [23]. This gene was first discovered and characterized in *M. truncatula* by RNA interference (RNAi) and in situ hybridization, which indicated a key role during nodule development, possibly by controlling nodule meristem function [23]. In *P. sativum*, *PsHAP2.1* showed low expression in roots and much higher expression in fully developed nodules [24]. In our condition, this gene was not expressed in very young seedlings (2-day-old) (Figure 1), and its expression was induced only in the presence of *Rhizobium*-derived signals in 5- and 12-day-old seedlings with an important increase between these two dates (Figure 2). Also worth noting is a significant decrease in expression under high nitrate concentration in 12-day-old seedlings (Figure 1).

*MtNPF7.6* encodes a nitrate transporter that belongs to the nitrate peptide family (NPF) that is specifically expressed in infected tissue. It has been discovered in *M. truncatula* where the encoded protein functions as a high-affinity transporter of nitrate specifically in the nodule vasculature [25]. This result indicates that this gene is not involved in early stages of nodulation process. Accordingly, in our conditions, an ortholog of *MtNPF7.6*, named *PsNPF7.1* [17], was not expressed in 2- and 5-day-old seedlings, irrespective of the condition, and showed an induction of expression in 12-day-old seedlings only in the presence of *Rhizobium* (Figure 1). The level of expression of this gene was by far higher when plants were fed with 1 mM NO_3_^−^ as if there was a synergistic effect between the low-nitrate signal and the *Rhizobium*-derived signal. It is worth noting, however, that this synergy was not operational under high nitrate concentration, which is a non-favorable condition for nodulation. 

#### 2.1.2. Markers of Molecular Response to Nitrate Signal 

There are two systems of nitrate transport functional in plants depending on exogenous nitrate concentration. At high nitrate availability (above 1 mM), nitrate transport occurs through a low-affinity transport system (LATS) that belongs to a family of proteins named NPF (NRT1/PTR Family) [26]. At low nitrate availability, nitrate transport occurs through a two-component high-affinity transport system (HATS) that belongs to the NAR2/NRT2 (NITRATE TRANSPORTER 2) family [27]. 

**Figure 3:**
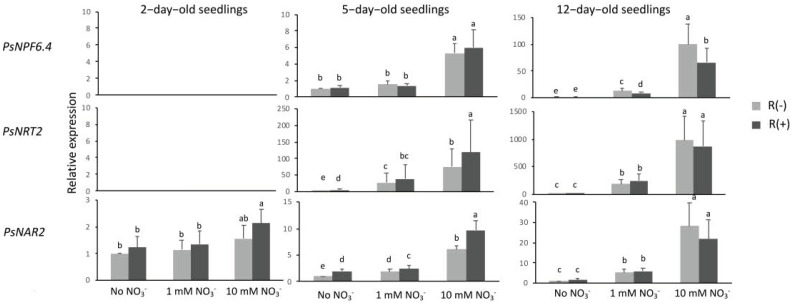
Relative expression of nitrate-inducible genes. The samples and the conditions used for RT-qPCR experiments were the same as those described in the legend of Figure 1. *PsNPF6.4*, NRT1/PTR Family 6.4, and *PsNRT2*/*PsNAR2*, the two component high-affinity nitrate transport system. Data presented are means of three independent experiments performed in triplicate. Error bars indicate SE. Letters denote significant differences at the 0.05 level (multiple comparisons).

In the present work we studied the regulation of *PsNPF6.4*, an ortholog of *M. truncatula* nitrate transporter *MtNPF6.7*, identified in one of our previous works as a nitrate-inducible gene [22]. In here, *PsNPF6.4* expression was not detected in young (2-day-old) seedlings in all conditions and in 12-day-old seedling in the absence of nitrate, but it was slightly detected in 5-day-old seedlings in the absence of nitrate (Figure 3). A slight level of expression was also observed when NO_3_^−^ was supplied at 1 mM, and a further increase in the expression was observed with the increase in exogenous nitrate concentration to 10 mM. An effect of the presence of *Rhizobium* was observed in 12-day-old seedlings when the induction by nitrate was the highest. This induction was counteracted by *Rhizobium*-derived signals resulting in a significant decrease in *PsNPF6.4* relative expression at both nitrate concentrations with almost 40% to 50% inhibition of the relative expression. 

Regulation of the expression of *PsNRT2* and *PsNAR2* encoding the two-component high-affinity transport system was studied. Similarly to *PsNPF6.4*, *PsNRT2* expression was not detected in young (2-day-old) seedlings in all conditions and in older (5- and 12-day-old) seedlings in the absence of nitrate (Figure 3). Expression of this gene was induced by nitrate at both stages, namely, 5 and 12 days of growth. The expression was much higher in older seedlings, as it was also nitrate concentration-dependent. The level of expression was dramatically higher when plants were fed 10 mM NO_3_^−^ than that when they were fed 1 mM NO_3_^−^. *PsNAR2* showed a constitutive expression as it was expressed at the three stages of development studied in the absence of nitrate (Figure 3). *PsNAR2* expression increased with seedling age as quantified by q-RT-PCR and visualized by the semi-quantitative PCR results obtained for the three periods of growth without NO_3_^−^ (Figure 2). Furthermore, *PsNAR2* proved to be nitrate-inducible, which was particularly evident in 5-day-old and 12-day-old seedlings, although it seemed to start in young (2-day-old) seedlings under 10 mM NO_3_^−^ (Figure 3). As to sensitivity to the presence of *Rhizobium*, an effect on the two-component high-affinity transport system was observed in 5-day-old seedlings. Unlike *PsNPF6.4* for which *Rhizobium*-derived signals exerted an inhibitory effect, *PsNRT2* and *PsNAR2* expressions were increased in infected plants in the presence and absence of nitrate; for instance, *PsNAR2* relative expression doubled in the absence of nitrate and increased by 40% in seedlings fed 10 mM NO_3_^−^. 

**Figure 4:**
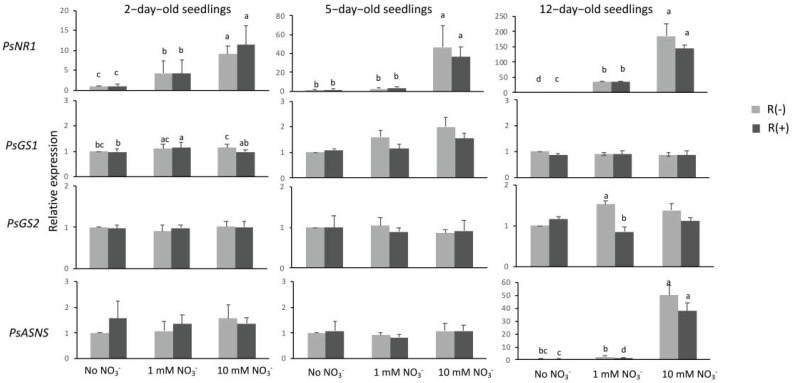
Relative expression of genes encoding enzymes of nitrate metabolism. The samples and the conditions used for RT-qPCR experiments were the same as those described in the legend of Figure 1. *PsNR1*, nitrate reductase; *PsGS1*, cytosolic glutamine synthetase; *PsGS2*, plastidic glutamine synthetase; *PsASNS*, asparagine synthetase. Data presented are means of three independent experiments performed in triplicate. Error bars indicate SE. Letters denote significant differences at the 0.05 level (multiple comparisons).

Once NO_3_^−^ has been taken up, it is reduced by nitrate reductase, an enzyme encoded by *NR1* that is known to be nitrate-inducible, as we have shown recently in *M. truncatula* [22]. In the present work, we found that in pea, also induction of *PsNR1* by nitrate held true, and it was observed at all three stages of development (Figure 4). The level of induction by nitrate increased with the increase in nitrate concentration and with the age of the seedlings; thus, the highest relative expression was observed in 12-day-old seedlings fed 10 mM NO_3_^−^ (Figure 4). The presence of *Rhizobium* in general seemed to have no effect on the expression of this gene with the exception of an increase in 12-day-old seedlings in the absence of NO_3_^−^ (Figure 2). 

Ammonium generated by the reduction of NO_3_^−^ and NO_2_^−^ is assimilated by glutamine synthetase. Expression of cytosolic *PsGS1* was measured in infected and uninfected seedlings supplied with NO_3_^−^ or not at the three stages of development studied in this experiment. The gene encoding GS1 was constitutively expressed at all stages and absolutely insensitive to *Rhizobium*-derived signals (Figure 4). It is worth noting the very high level of expression of this gene in pea seedlings (Figure 2). Expressions of two other genes encoding for two major enzymes of nitrogen primary metabolism, GS2 and asparagine synthetase (ASNS), were measured and, similarly to *PsGS1*, were mostly insensitive to *Rhizobium*-derived signals (Figure 4). 

### 2.2. Effect of Nitrate in Presence or Absence of Rhizobium on Phenotypic Traits 

An analysis of phenotypic traits was undertaken on plants grown in the hydroponics system in order to facilitate root system collection and architecture analysis, that is, root branching, total root length, and number of nodules. Plants were harvested after 17 days of growth (4-leaf stage) to allow for nodule development. Contrasting NO_3_^−^ concentrations were chosen in a preliminary experiment such that the lowest (5 mM) was both not N limiting during early post-germination and still allowing for nodulation, and the highest (14 mM) was high enough to show negative impacts on nodulation. Because cotyledons were severed (see Methods and Materials) in this experiment, our objective was to avoid N limitation symptoms that would bias our observation of potential interactions between the NO_3_^−^ signal per se and *Rhizobium*-derived signals.

#### 2.2.1. Root System Length and Branching 

Total root system length analyzed by the Kruskal–Wallis test did not indicate significant differences across treatments, neither for *Rhizobium* nor for nitrate, probably because the extreme values increased the dispersion (Table 1). Still, observation of the results showed that in uninfected plants, root system length was 5.5% shorter under 14 mM NO_3_^−^ treatment than that under 5 mM NO_3_^−^. Interestingly, in the presence of *Rhizobium*, the nitrate effect was the opposite. Root system length was 6.5% longer under 14 mM NO_3_^−^ than that under 5 mM NO_3_^−^ (Table 1).

In uninfected plants, root branching ratios were equivalent to 2.37 and 1.52 under 5 mM and 14 mM NO_3_^−^, respectively, which is a 36% decrease due to a lower number of tertiary roots per secondary root. In infected plants, statistical analysis of root branching indexes allowed for the detection of a trend (*p* < 0.06) for an effect of *Rhizobium*. Interestingly, in the presence of *Rhizobium*, the decrease in branching ratio observed under high nitrate was abolished, with branching ratios equivalent to 2.50 and 2.47 under 5 mM and 14 mM NO_3_^−^, respectively (Table 1).

#### 2.2.2. Nodules Number 

A nitrate effect was observed on nodulation in infected-plants (*p* < 10^−7^). As expected, the number of nodules decreased with the increase of exogenous nitrate concentration (*p* < 10^−7^) when plants were infected by *Rhizobium* (*p* < 10^−15^) (Table 1). Furthermore, among the residual nodules, a non-negligible number did not display the pinkish color specific to functional leghemoglobin (data not shown).

## 3. Discussion

The effect of nitrate on signaling processes initiated by *Rhizobium*-secreted Nod factors is a long-lasting topic of research to which a large number of works and publications has been dedicated. Furthermore, ammonium, when available as an N resource in the soil, results in the reduction of SNF [28]. On the contrary, the effect of Nod factors on signaling pathways initiated by nitrate acting as a signal molecule in plants has received, to our knowledge, no interest so far. However, there are several arguments that let us suppose that Rhizobia via secreted molecules, not only Nod factors, in the rhizosphere, would interfere with the nitrate signaling pathway and finally modify the response to nitrate signals (see Introduction). In the present work, this interaction was examined at two levels: (i) at the molecular level, namely, the regulation of gene expression by nitrate; and (ii) at the whole plant level, namely, the response of phenotypic traits to the nitrate signal. 

### 3.1. Effect of Rhizobium on Pea Seedling Response to NO_3_^−^ Signal at the Molecular (Transcriptional) Level

The effectiveness of *Rhizobium*-derived signals in our conditions was assessed by analyzing the expression of genes known to be involved in the common symbiosis-signaling pathway (CSSP) [29]. Stimulation of the CSSP by Nod factors was shown to result in the activation of a set of transcription factors, among which HAP2.1 and NSP1 orchestrate infection, nodule organogenesis, and autoregulation of nodulation (AON) [12]. In our conditions, *PsHAP2.1* was exclusively expressed in infected seedlings, while *PsNSP1* showed a constitutive expression, even in uninfected seedlings, but in 5- and 12-day-old seedlings its expression was up-regulated in infected seedlings compared to that in uninfected seedlings, indicating a specific effect of *Rhizobium*-derived signals. The third gene analyzed, *PsNPF7.1*, is the ortholog of *MtNPF7.6*, which is a gene that encodes, in *M. truncatula*, a high-affinity nitrate transporter known to be expressed specifically in nodule transfer cells, a structure occurring during advanced stages of nodule organogenesis [25]. Indeed, the CRISPR-Cas9 knockout mutation of this gene in *M. truncatula* resulted in developmental defects of the nodule vasculature [25]. Accordingly, in our conditions, *PsNPF7.1* was expressed only in 12-day-old infected seedlings. Wang et al. [25] proposed that MtNPF7.6 has been co-opted into a regulatory role in nodulation, functioning in nitrate uptake through nodule transfer cells to fine-tune nodule symbiosis in response to fluctuating environmental nitrate status. Our finding that *PsNPF7.1* expression was up-regulated by 1 mM NO_3_^−^ and down-regulated by 10 mM to its basal level of expression seems in agreement with this assertion. 

Altogether, the results of expressions of genes encoding PsHAP2.1, PsNSP1, and PsNPF7.1 show unequivocally that in our experimental conditions, infection of seedlings with *Rhizobium* was effective, and seedlings responded to *Rhizobium*-derived signals.

Similarly, the effectiveness of the NO_3_^−^ signal and its potential modulation by *Rhizobium*-derived signals was assessed by analyzing the expression of genes known to be NO_3_^−^-inducible. Chosen genes belong either to families of genes encoding transporters involved also in NO_3_^−^ signaling (PsNPF6.4 and NRT2/NAR2) or families of genes encoding enzymes of nitrate metabolism (NR1, GS1, GS2, and ASNS). 

*PsNPF6.4* is the one of the four close homologs of *A. thaliana AtNPF6.3* [17] that was thoroughly characterized and described as a transceptor because of its double role as a double affinity nitrate transporter and a nitrate signal sensor [30,31]. *PsNPF6.4* was also described as preferentially expressed in pea root [24]. *PsNPF6.4* exhibited low but still significant constitutive expression and proved to be also NO_3_^−^-inducible in a concentration-dependent manner. In 5- and particularly 12-day-old seedlings, its relative expression increased almost 10 and 100 times with the increase of NO_3_^−^ concentration from 1 mM to 10 mM NO_3_^−^, respectively. Although very low, we could see that the constitutive component of expression (in the absence of exogenous NO_3_^−^) was not sensitive to the presence of *Rhizobium*, while the NO_3_^−^-inducible component was negatively affected by *Rhizobium*-derived signals (40% to 50% inhibition) when the induction was at its highest level in 12-day-old seedlings at both exogenous NO_3_^−^ concentrations. Taken together, these two observations suggest that the *Rhizobium*-derived signal counteracted specifically the stimulating action of the NO_3_^−^ signal on *PsNPF6.4* expression, probably by interacting with the NO_3_^−^ signaling pathway. 

In *A. thaliana*, AtNRT2.1 and AtNAR2.1 were shown in heterologous expression systems, yeast and oocytes, to constitute a functional high-affinity nitrate transport system (HATS) [27]. AtNAR2.1 is essential in the HATS, as it has a role in targeting AtNRT2.1 to the plasma membrane and in forming a functional heteromere [32]. In planta, HATS has been shown to not only transport NO_3_^−^ at low concentrations (below 1 mM), but also to mediate NO_3_^−^ signaling, independently of NO_3_^−^ uptake, specifically root system branching through the lateral root response [33]. Under our conditions, *PsNRT2* appeared principally as NO_3_^−^-inducible in a concentration-dependent manner. *PsNAR2.1*, however, exhibited a more complex pattern of regulation compared to its partner. Not only did this gene show a constitutive expression in the absence of NO_3_^−^, but its expression was further simulated by NO_3_^−^ in a concentration-dependent manner. It is interesting to note that both components of the high-affinity transport system showed sensitivity to *Rhizobium*-derived signals in 5-day-old seedlings. The effect was more pronounced on *PsNAR2.1*, the relative expression of which doubled in the absence of nitrate (constitutive expression at 0 mM NO_3_^−^) and increased significantly in NO_3_^−^-fed seedlings (40% increase in seedlings fed 10 mM NO_3_^−^). To our knowledge, these results show for the first time alteration of the expression of genes encoding components of the HATS in legumes by *Rhizobium*-derived signals. Taken together, our results suggest that if HATS is involved in a *Rhizobium*-triggered process, it is unlikely to be in relation to NO_3_^−^ transport, since significant stimulation of *PsNAR2.1* expression was observed in infected seedlings in the absence of exogenous NO_3_^−^. 

Analyses of expressions of genes encoding enzymes of NO_3_^−^ metabolism, namely, nitrate reductase, cytosolic and plastidic glutamine synthetase isoforms, and asparagine synthetase, showed that they were either not sensitive or marginally affected by *Rhizobium*-derived signals. This observation strengthens the idea that if *Rhizobium* would interact via secreted signaling molecules with a NO_3_^−^-mediated process, it would do so with a component related to NO_3_^−^-signaling via transporters/sensors of the legume partner rather than with metabolic pathway of nitrate assimilation.

### 3.2. Effect of Rhizobium on Pea Seedling Response to NO_3_^−^ Signal at Root System Development Level 

Root system branching, in particular lateral root (LR) growth, is a sensitive and easily measurable trait to evaluate the response of plants to NO_3_^−^ [5]. In *A. thaliana*, the transfer of seedlings from high (10 mM) to low NO_3_^−^ (0.5 mM) resulted in increased root system branching as a result of increased LR length and enhanced LR appearance compared with plants remaining on 10 mM nitrate supply [27]. This effect of the NO_3_^−^ signal was abolished in mutants affected in at least one component of the HATS, namely, AtNAR2/AtNRT2. Both mutants showed the same inhibition of LR initiation on the newly developed primary root in response to low nitrate supply with a stronger phenotype for *atnar2.1-1* (depleted in NAR2) [27]. In our experiments in uninfected pea, we observed similar effects of NO_3_^−^ as in *Arabidopsis*; the root system branching ratio was higher in plants fed 5 mM NO_3_^−^ compared to those fed 14 mM NO_3_^−^ (fewer lateral roots of order 3 on lateral roots of order 2 under 14 mM NO_3_^−^), further supporting the idea that the NO_3_^−^ effect on root system branching would be conserved across species. Similar to our observation in *M. truncatula* [9], although not significant statistically, there was a consistent decrease in total root length in uninfected seedlings under 14 mM NO_3_^−^ compared to that under 5 mM NO_3_^−^. If these observations are consistent with the literature, the novelty of our work is the observation that these effects of the NO_3_^−^ signal were abolished in infected plants, probably as a result of the interaction of *Rhizobium*-derived signals with the NO_3_^−^ signal. The number of lateral roots of order 3 per lateral root of order 2 was higher in infected plants compared to uninfected plants under both NO_3_^−^ concentrations. This is counterintuitive, because one would expect fewer lateral roots on plants that develop nodules due to the alleged competition between lateral roots and nodule development. *Rhizobium*-counteracting effects on nitrate limitation of root branching and foraging allow for favorable management of trade-offs between nodules growth for nitrogen capture and root foraging for water and other soil nutrient uptake. It seems, however, that this inhibitory effect of *Rhizobium* on NO_3_^−^-signaling did not occur by targeting either NAR2 or NRT2, which were found to be essential for the effect of the NO_3_^−^ signal on root branching [27]. On the contrary, in infected pea plants, *PsNAR2* expression, as mentioned above, was even increased in infected compared to uninfected 5-day-old seedlings, thus suggesting that *Rhizobium*-derived signals target another component of the NO_3_^−^-signaling pathway in pea for regulating root branching.

## 4. Materials and Methods

### 4.1. Analysis of Molecular Traits

#### 4.1.1. Seedling Growth Conditions

For molecular analysis, *P. sativum* cv. Frisson plants were grown in a growth chamber (16 h days at 24 °C and 8 h nights at 10 °C) on neutral substrate, perlite, either with N-free MS solution or MS solution supplied with 1 mM or 10 mM KNO_3_ as described in [9] with or without *Rhizobium leguminosarum* P221 (OD_600_ = 0.08). For *Rhizobium* inoculation, a culture tube of the strain (brought to room temperature 24 h previous) was suspended in milliQ water (2.5 mL·L^−1^ solution), which resulted in a final concentration of 6.25 × 10^6^ cells·L^−1^ nutrient solution. This suspension was added after sowing on the top of the seedbed for the modalities with *Rhizobium*. An equivalent volume of milliQ water was added to the modalities without symbiotic bacteria. Roots from 10 plants in each condition were collected from 2, 5, or 12-day-old seedlings corresponding, respectively, to radicle emergence, cross-leaf emergence, and 2-leaf stage. Three independent biological repeats were performed.

#### 4.1.2. RNA Extraction and Reverse Transcription

Frozen roots were crushed in liquid nitrogen. Total RNAs were extracted from root powder with the Nucleospin^®^ RNA Plus kit (Macherey-Nagel, Düren, Germany) in accordance with the manufacturer’s instructions. RNA quality was checked using a Bioanalyser Instrument 2100 (Agilent Technologies, Santa Clara, CA, USA). One microgram of RNAs was treated by 0.5 U of DNase I (ThermoScientific, Waltham, MA, USA) and then used as a template for reverse transcription using the iScript Reverse Transcription Supermix kit (Bio-Rad, Hercules, CA, USA) according to the manufacturer’s protocol. 

#### 4.1.3. Real-Time Quantitative PCR

Real-time quantitative PCR was performed on a CFX96 Real-time Detection System (Bio-Rad) using primers designed with the eprimer3 tool (https://www.bioinformatics.nl/cgi-bin/emboss/eprimer3 accessed on 1 October 2021). The primers are listed in Table 2. Each reaction mix contained 2 µL diluted cDNA (1:8), 5 µL of SsoAdvanced™ Universal SYBR^®^ Green Supermix (Bio-rad, France), and 0.5 µM each primer, for a final volume of 10 µL. The following cycling conditions were applied: initial denaturation at 95 °C for 30 s, followed by 40 cycles at 95 °C for 10 s and 60 °C for 20 s. The specificity of the PCR amplification was checked with a heat-dissociation protocol after the final cycle of PCR. Each measurement was carried out with three independent biological replicates, using a triplicate PCR reaction for determining cycle threshold values. The normalization method was ΔΔct using actin and *β-*tubulin as reference genes [34].

#### 4.1.4. Semi Quantitative RT-PCR

For RT-PCR, cDNA amplification was carried out in a ThermalCycler T100 (Bio-Rad) with a standard protocol (Tm = 60 °C). Each reaction was performed using the DreamTaq Green DNA Polymerase (ThermoScientific), strictly following the manufacturer’s instructions, with 1 µM of each primer and 200 µM of dNTPs mix (Eurobio) in a total reaction mixture of 20 μL. The PCR consisted of a preliminary denaturation step of 2 min at 95 °C, followed by 30 cycles of 10 s at 95 °C, 30 s at 60 °C, and 20 s at 72 °C, and a final elongation step of 10 min at 72 °C. The PCR products were visualized on sight with DNA stain (Euromedex) and 2% agarose gel.

#### 4.1.5. Statistical Analysis

Statistical tests were carried out with RStudio software (Boston, MA, USA, version 1.2.1335). The conditions of normal distribution and homogeneity of variances were met, so we perform parametric tests (analysis of variance (ANOVA) followed by Tukey’ HSD test). Statistically significant differences were denoted by *p* < 0.05.

### 4.2. Analysis of Phenotypic Traits 

#### 4.2.1. Biological Material and Experimental Conditions

For phenotyping experiments, *Pisum sativum* cv. Frisson seeds were germinated on Whatman^®^ filter paper and soaked with demineralized water inside germination boxes (25 seeds per box; 18 × 12 × 5 cm). Seedlings were placed in culture 3 days after imbibition so that their radicle could reach the nutrient solution when transferred to the hydroponics system. As described above, a suspension of *Rhizobium leguminosarum* culture at a final inoculum concentration of 6.25 × 10^6^ cells·L^−1^ nutrient solution was added after sowing on the top of the seedbed for the modalities with *Rhizobium.* Plants were then cultivated in a hydroponics system with one pea per 2.2 L pot, covered with aluminum, filled with a regular nutrient solution adjusted to either 5 or 14 mM KNO_3_ with or without *Rhizobium leguminosarum*. The nutrient solution was oxygenated by bubbling air through two capillaries passing through the lid and held in place by Terostat^®^ putty. In order to limit nitrogen supply from the cotyledons during the first 10 days of growth [19] and to cause more contrasted response between nitrate treatments, cotyledons and the integuments were severed with a scalpel after the first leaf was deployed. A random position was assigned to each plant in the greenhouse where temperature was maintained between 10 °C at night and 24 °C during the day. Neon lights and automatic shading helped simulate long days and limit the light intensity in strong sunshine.

#### 4.2.2. Data Collection

Pea plants were harvested at the 4-leaf stage (17 days of culture), aerial parts were cut, and roots were washed with tap water and spread on A4 plastic transparencies. Nodules were counted before scanning the root system (EPSON Perfection V700, Regent Instrument Canada Inc.).

Branching Ratio index was calculated as indicated in Equation (1).

Branching Ratio = Tertiary roots number/secondary roots number(1)

Root system architecture analysis, root length, and branching were performed with WinRhizoTM 2012b software (Regent Instruments Canada Inc.).

#### 4.2.3. Statistics

The database combining the analysis results by WinRhizoTM and the various measurements obtained was carried out using MicroSoft Excel^®^ software. Statistical analyses and graphs were conducted with R software^®^ (version 3.6.0) using the RStudio^®^ interface (R Core Development Team 2014). Two-way ANOVA and Tukey tests were performed to determine significant differences within factorial modalities, when the normality and homoscedasticity assumptions were validated. The natural logarithm ln(x + 1) was applied to the data when the residuals did not follow a Normal distribution or in the case of heteroskedasticity in the data. In the case of non-normal residuals distribution or unequal variances, even after data transformation, the non-parametric Kruskal–Wallis test was used.

## 5. Conclusions

The nitrate-inhibiting effect on nodulation by counteracting signaling processes initiated by *Rhizobium*-secreted Nod factors has aroused great interest, and a wealth of literature is available on this subject. NO_3_^−^ triggers AON (Autoregulation of Nodulation), a regulatory mechanism that controls the number of nodules by inducing the production of a Q signal molecule that acts locally. The perception of the Q molecule leads to the production of an inhibiting factor that suppresses further nodulation events [35,36]. This is well illustrated in the present work by the observed decrease in the number of nodules under 14 mM NO_3_^−^ compared to that under 5 mM. In contrast, to our knowledge, no interest has been given so far to the effect of Rhizobia in the nitrate signaling pathway, particularly at early stages of post-germination growth and seedling establishment when NO_3_^−^ was shown to be determinant in shaping root system architecture (see Introduction). The objective of the present work was to tackle this issue, since these two components of the rhizosphere, namely, nitrate and *Rhizobium*-derived signals, are important determinants of root architecture, as they are both involved in trade-offs between nodules and lateral root formation. At the molecular level, expressions of two major players involved in NO_3_^−^ transport and sensing–signaling, namely, *PsNPF6.4*, the ortholog of *A. thaliana* transceptor *AtNPF6.3*, and *PsNRT2.1*-*PsNAR2.1*, genes encoding the two-component high-affinity nitrate transport system (HATS), were altered in the presence of *Rhizobium*. At the phenotypic level, the novelty of our work is to show that in the presence of *Rhizobium*, the characteristic limitation of root foraging and branching in response to NO_3_^−^ supply was abolished. At each NO_3_^−^ treatment, the number of tertiary roots was higher in infected compared to uninfected peas, thus indicating that the *Rhizobium* effect allows for favorable management of trade-offs between nodule growth for nitrogen capture and root foraging for water and other nutrient uptake. In conclusion, our work brings evidence that the presence of *Rhizobium*, likely via secreted signaling molecules in the rhizosphere, interfere with NO_3_^−^-mediated processes in pea.

The present work constitutes a launching pad for in-depth investigations (i) to assess at the whole transcriptome level to what extent nitrate signaling is altered by *Rhizobium*-derived signaling molecules; (ii) to uncover genes whose expression is targeted by the signals produced by *Rhizobium*; and (iii) to determine what are the related consequences on the root system architecture during the crucial period of seedling establishment. Moreover, the outcome of these basic approaches can be used to produce molecular tools for breeding pea genotypes able to develop a deep-foraging and branched root system, more competitive at phenotypic and molecular levels for the absorption of soil NO_3_^−^ during seedling establishment without jeopardizing nodulation. 

## Figures and Tables

**Table 1:** Means (± SE) of phenotypic traits of pea plants grown in hydroponics with or without *Rhizobium* and fed nutrient solution with nitrate concentrations of 5 or 14 mM nitrate. Letters (a, b, c) correspond to the significant differences detected by the Tukey test between the experimental conditions for each variable.

***Rhizobium***	**−**		**+**	
**Nitrate Concentration**	**5 mM**	**14 mM**	**5 mM**	**14 mM**
Nodules Number	8 ± 2b	1 ± 1a	65 ± 4c	42 ± 6c
Total Root Length (cm)	842 ± 23	796 ± 50	794 ± 44	850 ± 11
Secondary Roots Number	49 ± 3	55 ± 3	53 ± 2	45 ± 1
Tertiary Roots Number	117 ± 14	85 ± 16	134 ± 18	112 ± 13
Branching Ratio	2.37 ± 0.30	1.52 ± 0.23	2.50 ± 0.27	2.47 ± 0.26
Sample size	10	8	9	9

**Table 2:** List of primer sequences used for PCR analysis.

Gene ID	Gene Name		Primers
Psat5g198720	*β-tubulin*	Forward	CAGAACAAGAACTCGTCATACT
		Reverse	AGCCTTCCTCCTGAACATA
Psat5g063760	*Actin*	Forward	CTAAGGGTGAATATGATGAGTCTGG
		Reverse	GAGACACCAAAAAAGCAACCACATC
Psat0s741g0280	*PsNSP1*	Forward	AAGCATTGACAAACCAGCGT
		Reverse	ATGTTGCCCCTTCCACCATA
Psat6g092920	*PsHAP2.1*	Forward	GCTGAGCCGTACAATCGTTT
		Reverse	AAGTTGGTCCGTCGTCAGAT
Psat6g242600	*PsNPF7.1*	Forward	TGCGGAAAATGGGATGTTGT
		Reverse	CTCCACGGATTGTTTCGAGTC
Psat2g178360	*PsNPF6.4*	Forward	ATTGCAGTGGTGTTTGTGGC
		Reverse	ACGACTTTGATTGCCGCTTT
Psat7g149120	*PsNRT2*	Forward	TGCCTTTTTATCCTGGTTGG
		Reverse	GTAAAGCAGCGCAAAAATCC
Psat4g061680	*PsNAR2*	Forward	GGACGATCTCTCAAGGGACA
		Reverse	TATCAGCATTCGTGCTTTGC
Psat2g077040	*PsNR1*	Forward	GTCGACGGAAAATTGACGAT
		Reverse	CCTACCGGGCCTTTTACTTC
Psat5g120440	*PsGS1*	Forward	TTTGCCGGCATCAACATCAG
		Reverse	AGCACCATTCCAATCACCCT
Psat0s690g0040	*PsGS2*	Forward	ACGAGGTAATCAAGAAGGCGA
		Reverse	ATTGAGCTTCCACGGTTTGC
Psat5g153000	*PsASNS*	Forward	TCACTACGATAAGGGCTGCAA
		Reverse	GCTTTGATCTTGCGGCATGT

## References

[1] Galloway J.N., Cowling E.B. (2021). Reflections on 200 years of Nitrogen, 20 years later: This article belongs to Ambio′s 50th Anniversary Collection. Theme: Eutrophication. Ambio.

[2] Hirsch A.M., Lum M.R., Downie J.A. (2001). What makes the rhizobia-legume symbiosis so special?. Plant Physiol..

[3] Brooker R.W., Bennett A.E., Cong W.F., Daniell T.J., George T.S., Hallett P.D., Hawes C., Iannetta P.P., Jones H.G., Karley A.J. (2015). Improving intercropping: A synthesis of research in agronomy, plant physiology and ecology. New Phytol..

[4] Annicchiarico P., Nazzicari N., Notario T., Martin C.M., Romani M., Ferrari B., Pecetti L. (2021). Pea Breeding for Intercropping with Cereals: Variation for Competitive Ability and Associated Traits, and Assessment of Phenotypic and Genomic Selection Strategies. Front. Plant Sci..

[5] Zhang H., Forde B.G. (2000). Regulation of Arabidopsis root development by nitrate availability. J. Exp. Bot..

[6] Zhang H., Rong H., Pilbeam D. (2007). Signalling mechanisms underlying the morphological responses of the root system to nitrogen in *Arabidopsis thaliana*. J. Exp. Bot..

[7] Wang Y.Y., Cheng Y.H., Chen K.E., Tsay Y.F. (2018). Nitrate Transport, Signaling, and Use Efficiency. Annu. Rev. Plant Biol..

[8] Hu B., Jiang Z., Wang W., Qiu Y., Zhang Z., Liu Y., Li A., Gao X., Liu L., Qian Y. (2019). Nitrate-NRT1.1B-SPX4 cascade integrates nitrogen and phosphorus signalling networks in plants. Nat. Plants.

[9] Pellizzaro A., Clochard T., Cukier C., Bourdin C., Juchaux M., Montrichard F., Thany S., Raymond V., Planchet E., Limami A.M. (2014). The nitrate transporter MtNPF6.8 (MtNRT1.3) transports abscisic acid and mediates nitrate regulation of primary root growth in Medicago truncatula. Plant Physiol..

[10] Zhang H., Jennings A., Barlow P.W., Forde B.G. (1999). Dual pathways for regulation of root branching by nitrate. Proc. Natl. Acad. Sci. USA.

[11] Herrbach V., Remblière C., Gough C., Bensmihen S. (2014). Lateral root formation and patterning in Medicago truncatula. J. Plant Physiol..

[12] Oldroyd G.E., Downie J.A. (2008). Coordinating nodule morphogenesis with rhizobial infection in legumes. Annu. Rev. Plant Biol..

[13] Oláh B., Brière C., Bécard G., Dénarié J., Gough C. (2005). Nod factors and a diffusible factor from arbuscular mycorrhizal fungi stimulate lateral root formation in Medicago truncatula via the DMI1/DMI2 signalling pathway. Plant J..

[14] Phillips D.A., Torrey J.G. (1972). Studies on cytokinin production by *Rhizobium*. Plant Physiol..

[15] Ernstsen A., Sandberg G., Crozier A., Wheeler C.T. (1987). Endogenous indoles and the biosynthesis and metabolism of indole-3-acetic acid in cultures of Rhizobium phaseoli. Planta.

[16] Roy S., Liu W., Nandety R.S., Crook A., Mysore K.S., Pislariu C.I., Frugoli J., Dickstein R., Udvardi M.K. (2020). Celebrating 20 Years of Genetic Discoveries in Legume Nodulation and Symbiotic Nitrogen Fixation. Plant Cell.

[17] Gu B., Chen Y., Xie F., Murray J.D., Miller A.J. (2022). Inorganic Nitrogen Transport and Assimilation in Pea. Genes.

[18] Kreplak J., Madoui M.A., Cápal P., Novák P., Labadie K., Aubert G., Bayer P.E., Gali K.K., Syme R.A., Main D. (2019). A reference genome for pea provides insight into legume genome evolution. Nat. Genet..

[19] Voisin A.S., Bourion V., Duc G., Salon C. (2007). Using an ecophysiological analysis to dissect genetic variability and to propose an ideotype for nitrogen nutrition in pea. Ann. Bot..

[20] Pellizzaro A., Clochard T., Planchet E., Limami A.M., Morère-Paven M.C. (2015). Identification and molecular characterization of Medicago truncatula NRT2 and NAR2 families. Physiol. Plant..

[21] Zang L., Morère-Paven M.C., Clochard T., Porcher A., Satour P., Mojović M., Vidović M., Limami A.M., Montrichard F. (2020). Nitrate inhibits primary root growth by reducing accumulation of reactive oxygen species in the root tip in Medicago truncatula. Plant Physiol. Biochem..

[22] Zang L., Tarkowski Ł., Morère-Paven M.C., Zivy M., Balliau T., Clochard T., Bahut M., Balzergue S., Pelletier S., Landès C. (2022). The Nitrate Transporter MtNPF6.8 is a Master Sensor of Nitrate Signal in the Primary Root Tip of *Medicago truncatula*. Front. Plant Sci..

[23] Combier J.P., Frugier F., de Billy F., Boualem A., El-Yahyaoui F., Moreau S., Vernié T., Ott T., Gamas P., Crespi M. (2006). MtHAP2-1 is a key transcriptional regulator of symbiotic nodule development regulated by microRNA169 in Medicago truncatula. Genes Dev..

[24] Alves-Carvalho S., Aubert G., Carrère S., Cruaud C., Brochot A.L., Jacquin F., Klein A., Martin C., Boucherot K., Kreplak J. (2015). Full-length de novo assembly of RNA-seq data in pea (*Pisum sativum* L.) provides a gene expression atlas and gives insights into root nodulation in this species. Plant J..

[25] Wang Q., Huang Y., Ren Z., Zhang X., Ren J., Su J., Zhang C., Tian J., Yu Y., Gao G.F. (2020). Transfer cells mediate nitrate uptake to control root nodule symbiosis. Nat. Plants.

[26] Léran S., Varala K., Boyer J.C., Chiurazzi M., Crawford N., Daniel-Vedele F., David L., Dickstein R., Fernandez E., Forde B. (2014). A unified nomenclature of NITRATE TRANSPORTER 1/PEPTIDE TRANSPORTER family members in plants. Trends Plant Sci..

[27] Orsel M., Chopin F., Leleu O., Smith S.J., Krapp A., Daniel-Vedele F., Miller A.J. (2006). Characterization of a two-component high-affinity nitrate uptake system in Arabidopsis. Physiology and protein-protein interaction. Plant Physiol..

[28] Schulze J., Liese R., Ballesteros G., Casieri L., Salinas G., Cabeza R.A. (2020). Ammonium acts systemically while nitrate exerts an additional local effect on Medicago truncatula nodules. Plant Sci..

[29] Mergaert P., Kereszt A., Kondorosi E. (2020). Gene Expression in Nitrogen-Fixing Symbiotic Nodule Cells in *Medicago truncatula* and other nodulating plants. Plant Cell.

[30] Ho C., Lin S., Hu H., Tsay Y. (2009). CHL1 functions as a nitrate sensor in plants. Cell.

[31] Krouk G., Lacombe B., Bielach A., Perrine-Walker F., Malinska K., Mounier E., Hoyerova K., Tillard P., Leon S., Ljung K. (2010). Nitrate-regulated auxin transport by NRT1.1 defines a mechanism for nutrient sensing in plants. Dev. Cell.

[32] Yong Z., Kotur Z., Glass A.D. (2010). Characterization of an intact two-component high-affinity nitrate transporter from Arabidopsis roots. Plant J..

[33] Little D.Y., Rao H., Oliva S., Daniel-Vedele F., Krapp A., Malamy J.E. (2005). The putative high-affinity nitrate transporter NRT2.1 represses lateral root initiation in response to nutritional cues. Proc. Natl. Acad. Sci. USA.

[34] Gallardo K., Besson A., Klein A., le Signor C., Aubert G., Henriet C., Térézol M., Pateyron S., Sanchez M., Trouverie J. (2019). Transcriptional Reprogramming of Pea Leaves at Early Reproductive Stages. Front. Plant Sci..

[35] Nishida H., Suzaki T. (2018). Nitrate-mediated control of root nodule symbiosis. Curr. Opin. Plant Biol..

[36] Nishida H., Ito M., Miura K., Kawaguchi M., Suzaki T. (2020). Autoregulation of nodulation pathway is dispensable for nitrate-induced control of rhizobial infection. Plant Signal. Behav..

